# Herpes simplex virus-1 encephalitis induced by chemoradiotherapy and steroids in an esophageal cancer patient: a case report

**DOI:** 10.1186/s12885-016-2255-8

**Published:** 2016-03-17

**Authors:** Masaaki Saito, Hirokazu Kiyozaki, Tamotu Obitsu, Hirofumi Imoto, Yusuke Taniyama, Osamu Takata, Toshiki Rikiyama

**Affiliations:** Department of Surgery, Saitama Medical Center, Jichi Medical University, 1-847 Amanuma-cho, Omiya-ku, Saitama, 330-8503 Japan

**Keywords:** HSV-1, Encephalitis, Chemoradiotherapy, Esophageal cancer

## Abstract

**Background:**

Systemic chemotherapy combined with steroids used as prophylactic antiemetics have been reported to induce immunosuppression. Further, herpes simplex virus-1 (HSV-1) infection has been reported to occur in patients with small cell carcinomas after chemoradiotherapy that includes brain irradiation. Here, we report a case of HSV-1 encephalitis that occurred in a patient undergoing chemoradiotherapy for advanced esophageal cancer.

**Case presentation:**

A 77-year-old woman received chemoradiotherapy (5-fluorouracil, 700 mg/m^2^; cisplatin, 70 mg/m^2^; and radiotherapy, 60 Gy in total) for stage III esophageal cancer. The total radiation dose was administered concurrently with the first two courses of chemotherapy, together with dexamethasone as a prophylactic antiemetic. Two days before completion of the fourth course of chemotherapy, the patient developed acute neurological symptoms of disorientation, clouding of consciousness, and fever. T2-weighted magnetic resonance imaging showed a high intensity area in the bilateral temporal lobes and insular cortex. Furthermore, DNA PCR testing of cerebrospinal fluid showed clear positivity for HSV-1 DNA, and the patient was diagnosed with herpetic encephalitis. Intravenous administration of acyclovir for 3 weeks led to gradual improvement of consciousness, and the patient was able to respond to verbal cues.

**Conclusion:**

In advanced esophageal cancer patients, standard treatment involves chemoradiotherapy and surgery. However, primary infection with or reactivation of endogenous latent HSV-1 in the brain cortex during chemoradiotherapy combined with administration of a steroid may compromise the benefits of treatment.

## Background

Esophageal cancer patients are very likely to undergo chemotherapy and radiotherapy as definitive chemoradiotherapy for advanced esophageal cancer is a widely accepted standard treatment, with a combination of 5-fluorouracil (5-FU) and cisplatin with concurrent irradiation (50–60 Gy total dose) being a standard regimen [1, 2].

Herpes simplex virus (HSV) is a well-characterized double-stranded DNA virus that can latently infect the spinal, trigeminal, and sacral cord ganglia. One subtype of the virus, HSV type 1 (HSV-1) commonly infects the trigeminal ganglia and may reactivate and spread from there to result in herpes labialis, stomatitis, keratitis, or encephalitis.

Herpes simplex encephalitis (HSE) accounts for 10–20 % of encephalitis cases and is particularly noted in cases of sporadic encephalitis, the annual morbidity rate of which is 2–4 individuals per million [3–5]. HSV-1 is responsible for 95 % of all cases of HSE, and it is estimated that approximately 70–80 % of these occurrences are caused by reactivation of latent virus or re-infection, while the remaining cases are due to primary infection. Fatigue, trauma, and stress that weaken the host’s immune system can lead to reactivation of the latent virus. Moreover, systemic chemotherapy along with steroids used as prophylactic antiemetics may also induce immunosuppression.

Only a few case reports of HSE following chemotherapy or steroid therapy in cancer patients exist [6]. Herein, we report a case of HSV-1 encephalitis that occurred during chemoradiotherapy in a patient with advanced esophageal cancer.

## Case presentation

A 77-year-old woman had been suffering from dysphagia for 2 months prior to hospitalization. She was diagnosed with stage III esophageal cancer at a local hospital and was referred to our hospital for further treatment. Esophagogastroduodenoscopy showed a type 2 tumor in the lower intrathoracic esophagus. Enhanced computed tomography showed wall thickening and ambient lymphadenopathy. She received chemoradiotherapy (5-FU, 700 mg/m^2^; cisplatin, 70 mg/m^2^; and radiotherapy, 60 Gy in total) every 28 days. The total irradiation dose to the mediastinum was administered concomitantly with two courses of chemotherapy, combined with dexamethasone as a prophylactic antiemetic. Partial remission after two courses of chemoradiotherapy was achieved and the residual esophageal tumor was minimal.

However, 2 days before completion of the fourth course of chemotherapy, the patient developed acute neurological symptoms of disorientation, clouding of consciousness, and fever. At the onset, leukocyte count was 2020, and the lymphocyte count had decreased to 120/mm3. Serum squamous cell carcinoma antigen was 1.9 ng/mL and the remaining serological parameters were within normal ranges. Blood culture results were negative, and chest and abdominal radiography findings were unremarkable.

**Fig. 1:**
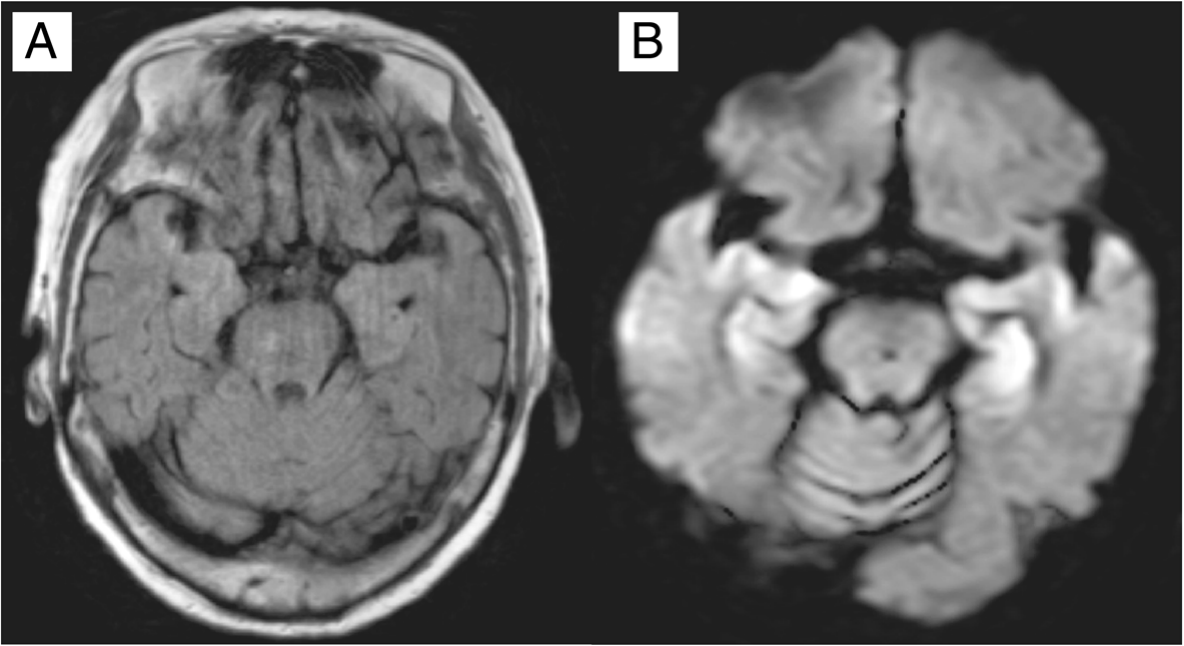
Magnetic resonance imaging of the brain upon onset of symptoms. **a** Coronal T2-weighted magnetic resonance imaging of the brain revealed high intensity areas bilaterally in the temporal lobes. **b** Diffusion-weighted imaging revealed enhanced high intensity areas corresponding to the bilateral temporal lobes

**Fig. 2:**
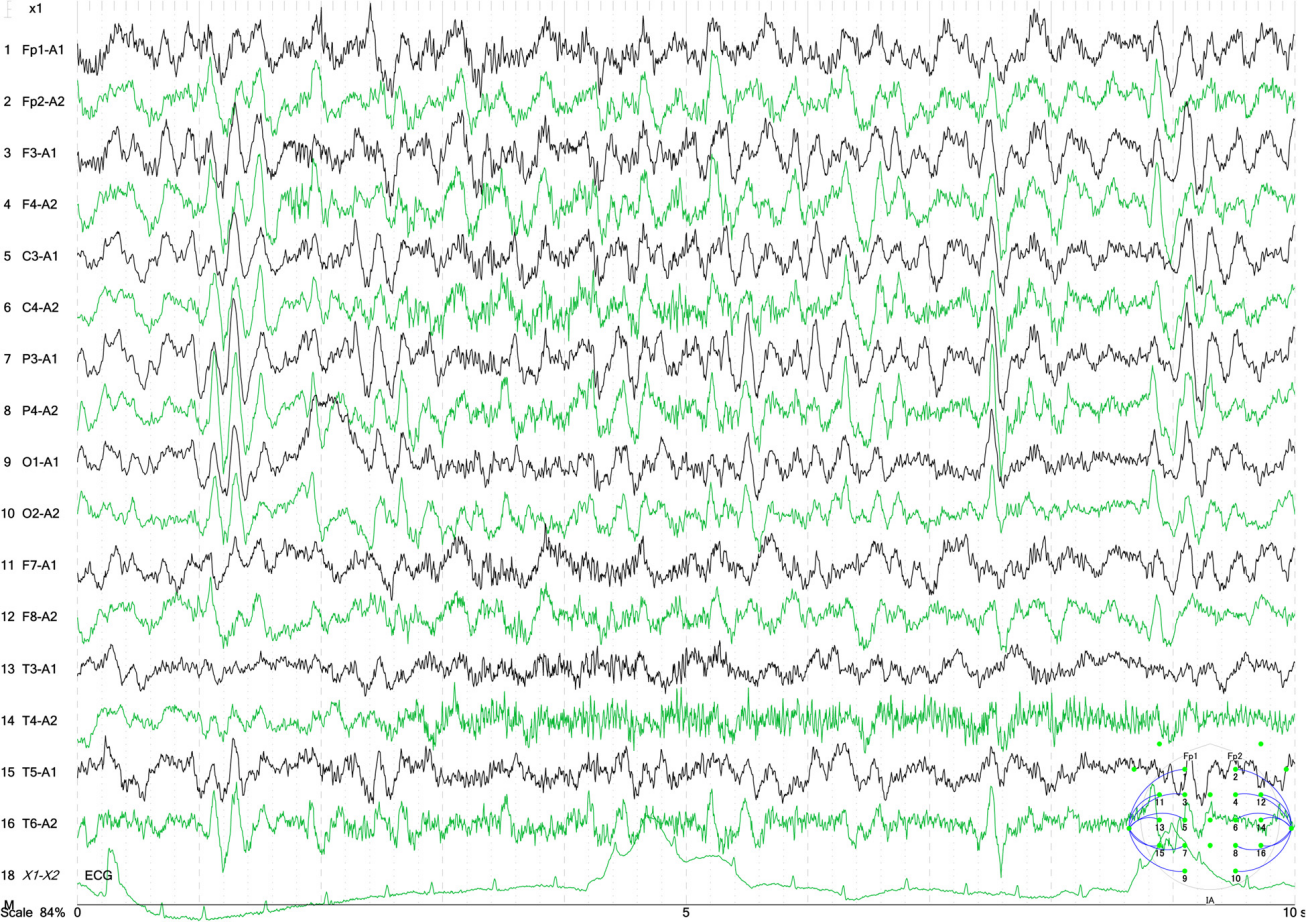
Electroencephalogram upon onset of symptoms. An electroencephalogram showed a diffuse sharp wave–slow wave composition wave

A computed tomography (CT) scan of the brain at the onset of symptoms revealed only multiple small low-density areas dispersed around a cerebral hemisphere, which were remnants of an earlier cerebral infarction. Coronal T2-weighted magnetic resonance imaging (MRI) of the brain revealed bilateral high intensity areas in the temporal lobes. Diffusion-weighted imaging revealed enhanced high intensity areas corresponding to the bilateral temporal lobes (Fig. 1). These findings strongly suggested acute encephalitis. An electroencephalogram showed a diffuse sharp wave–slow wave composition wave (Fig. 2).

Examination of cerebrospinal fluid showed no occurrence of pleocytosis of mononuclear cells, with only two monocytes and eight erythrocytes being identified in 3 μL of CSF. Furthermore, CSF glucose and protein were normal. However, the DNA PCR consensus herpes test showed clear positivity for HSV-1 DNA. Based on these findings, we arrived at a diagnosis of acute HSV-1 encephalitis by endogenous viral reactivation in an immunocompromised patient.

With intravenous administration of acyclovir for 3 weeks, the patient’s state of consciousness gradually improved, as she regained the ability to understand and respond to simple instructions. She was subsequently transferred to another hospital, where she is currently being treated. To date, her cancer has not recurred and, although she is confined to a wheelchair and fed by tube, she remains capable of responding to simple verbal cues.

## Discussion

The overall annual incidence of HSE is estimated at 2–4 individuals per million. An immunosuppressive state is thought to contribute to reactivation of latent HSV, and yet it is not expected to affect the incidence rate of HSE, although the severity of this disease is likely to be worse in the immunocompromised [7]. However, comorbidities of cancer, chemotherapy, radiotherapy, steroid therapy, and other disorders are known to elevate the incidence of HSE by decreasing cell-mediated immunity [5, 8].

Chemoradiotherapy for esophageal cancer is indicated for patients with resectable cancer who cannot tolerate surgery and those with unresectable stage T4 cancer or lymph node metastasis that is confined to one particular area. A phase II clinical study on the standard chemoradiotherapy regimen (FP therapy [5-FU, 1,000 mg/m^2^; cisplatin, 75 mg/m^2^] plus radiotherapy, 50.4 Gy) was conducted in patients with esophageal cancer at clinical stages II/III (excluding those with stage T4 cancer) in Japan [2]. Despite the favorable outcomes observed (complete response rate of 70 % and a 3-year survival rate of 63.8 %), acute toxicity was slightly increased. The toxicity of chemotherapy, especially cisplatin, includes nausea and vomiting in the acute phase. When such highly emetogenic drugs are administered, it is recommended that the following three-drug combination be included: oral administration of the neurokinin -1 receptor antagonist aprepitant at 125 mg, a 5-hydroxytryptamine-3 receptor antagonist, and dexamethasone at 12 mg [9–11].

In the present case, the patient received four courses of FP therapy and radiotherapy to the mediastinum. Additionally, steroids were also administered to prevent emesis. While systemic chemotherapy has been reported to induce suppression of systemic immunity, it is assumed that administration of steroids further contributed to the patient’s immunosuppression. Consistent with this, a decreased peripheral lymphocyte count was also observed.

Graber et al. summarized prior reports of cancer patients subsequently diagnosed with HSE, including patients from their own academic cancer center over a 12-year period. He reported that 19 cancer patients receiving chemotherapy developed HSE in the past 12 years [6], indicating a higher than expected incidence of HSE in this population. The cohort included 11 patients with brain tumors, three with lung cancer, two with breast cancer and one patient each with malignant lymphoma, multiple myeloma and renal cancer. Patients received various chemotherapeutic agents, and brain radiation was concomitantly administered in 13 of these individuals. Of the total cohort, two patients survived and 15 died of herpes encephalitis. Follow up data were incomplete for the remaining two individuals. There have been no reports of this disease occurring during chemotherapy for esophageal cancer, and the present case is thus the first reported case. This patient received systemic chemotherapy and directed radiation to the mediastinum; however, the specific relationship between development of HSE and mediastinal radiation remains unclear.

Because HSE is generally difficult to diagnose in cancer patients, it is assumed that there are patients who remain without a definitive diagnosis and experience unfavorable outcomes. The disease is often difficult to differentiate from brain metastasis, paraneoplastic syndrome, and cerebral infarction as a manifestation of Trousseau’s syndrome [12, 13]. Although fever, recurrent syncopal attacks and disorientation were observed in the present case, a clinical diagnosis was difficult to make. When cancer patients experience progressive neurological symptoms with evidence of inflammation, it is prudent to actively suspect a comorbidity of HSE, with brain metastasis also taken into consideration.

Many studies have been conducted regarding various diagnostic procedures for this disease [12, 13]. MRI depicts edematous changes due to inflammation as normal intensity areas on T1-weighted images and high intensity areas on T2-weighted images in the cortices of the bilateral temporal lobes, white matter, and insular cortex. MRI should allow for earlier diagnosis than a CT scan. In the present case, although no apparent finding was obtained by CT scan, MRI revealed mildly high intensity areas in the medial cortices of the bilateral temporal lobes and insular cortex on fluid-attenuated inversion recovery images.

The examination of cerebrospinal fluid of HSE patients generally reveals elevated cerebrospinal fluid pressure, cytosis with lymphocytic predominance, and increased protein. The glucose concentration is often normal. Erythrocytes or xanthochromia may be also detected in some cases [14].

When HSV-DNA is detected in the cerebrospinal fluid via PCR, a definitive diagnosis can be made; however, a negative result does not rule out HSE [15–17]. In the present case, PCR was indeed positive for HSV-DNA, leading to the definitive diagnosis.

Electroencephalograms show abnormalities in almost all cases of HSE. Focal abnormalities are found in many cases, whereas periodic lateralized epileptic discharges, which are considered to be relatively characteristic to HSE, are found in approximately 30 % of cases. The present case showed a diffuse sharp-and-slow-wave complex.

Since the advent of the use of acyclovir for the treatment of HSE, mortality has markedly decreased from 70 to 7.1–28 % [4–6, 18]. As a general guideline, acyclovir is intravenously infused at a dose of 10 mg/kg three times a day for 14 days or more [19, 20]. For the treatment of convulsive seizures and cerebral edema, diazepam, midazolam, phenytoin, or other agents are used. In order to treat cerebral edema, glyceol, mannitol, and steroids are recommended. The mechanisms of action of corticosteroids are assumed to include the reduction of cerebral edema and the inhibition of secretion of proinflammatory cytokines. In the present case, while acyclovir was administered for 3 weeks, the patient received methylprednisolone for 3 days at a dose of 1,000 mg/day to prevent cerebral edema and phenobarbital for 4 weeks at a dose of 100 mg/day to prevent convulsions. Although the prognosis of this disease has traditionally been extremely poor, our patient recovered. She was subsequently transferred to another hospital, where she is currently being treated.

## Conclusions

Any esophageal cancer patient who undergoes chemoradiotherapy and has subsequent neurologic decline should be evaluated for HSE. Furthermore, patients undergoing chemotherapy should be monitored, given the possibility of latent HSV-1 reactivation. When HSE is suspected, we recommend that antiviral therapy commence immediately, as this may prove lifesaving while the diagnosis is being confirmed.

## Consent

Written informed consent was obtained from the patient’s next-of-kin for publication of this case report and any accompanying images. A copy of the written consent is available for review by the Editor of this journal.

## Availability of data and materials

The datasets supporting the conclusions of this article are included within the article.

## Abbreviations

HSV: Herpes simplex virus
DNA: Deoxyribonucleic acid
HSE: Herpes simplex encephalitis
CT: Computed tomography
MRI: Magnetic resonance imaging
PCR: Polymerase chain reaction
